# Quinoxalin-2-yl *m*-tolyl ether

**DOI:** 10.1107/S1600536808026822

**Published:** 2008-08-23

**Authors:** Nor Duha Hassan, Hairul Anuar Tajuddin, Zanariah Abdullah, Seik Weng Ng

**Affiliations:** a Department of Chemistry, University of Malaya, 50603 Kuala Lumpur, Malaysia

## Abstract

The dihedral angle between the two aromatic ring systems in the title compound, C_15_H_12_N_2_O, is 79.4 (1)°. The angle at the O atom is widened to 116.93 (9)°.

## Related literature

The title compound exhibits fluorescence; see: Abdullah (2005 ▶); Kawai *et al.* (2001 ▶); Mohd Salleh *et al.* (2007 ▶).
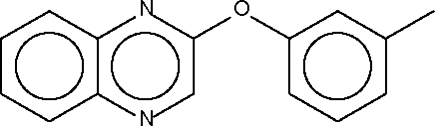


## Experimental

### 

#### Crystal data

C_15_H_12_N_2_O
*M* *_r_* = 236.27
Monoclinic, 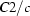

*a* = 18.5958 (5) Å
*b* = 7.0710 (1) Å
*c* = 19.4821 (5) Å
β = 112.487 (1)°
*V* = 2366.94 (9) Å^3^
*Z* = 8
Mo *K*α radiation
μ = 0.09 mm^−1^
*T* = 100 (2) K
0.40 × 0.15 × 0.10 mm

#### Data collection

Bruker SMART APEX diffractometer
Absorption correction: none
8036 measured reflections
2708 independent reflections
2179 reflections with *I* > 2σ(*I*)
*R*_int_ = 0.025

#### Refinement

*R*[*F* ^2^ > 2σ(*F* ^2^)] = 0.040
*wR*(*F* ^2^) = 0.112
*S* = 1.04
2708 reflections
164 parameters
H-atom parameters constrained
Δρ_max_ = 0.29 e Å^−3^
Δρ_min_ = −0.26 e Å^−3^

### 

Data collection: *APEX2* (Bruker, 2007 ▶); cell refinement: *SAINT* (Bruker, 2007 ▶); data reduction: *SAINT*; program(s) used to solve structure: *SHELXS97* (Sheldrick, 2008 ▶); program(s) used to refine structure: *SHELXL97* (Sheldrick, 2008 ▶); molecular graphics: *X-SEED* (Barbour, 2001 ▶); software used to prepare material for publication: *publCIF* (Westrip, 2008 ▶).

## Supplementary Material

Crystal structure: contains datablocks global, I. DOI: 10.1107/S1600536808026822/bt2770sup1.cif

Structure factors: contains datablocks I. DOI: 10.1107/S1600536808026822/bt2770Isup2.hkl

Additional supplementary materials:  crystallographic information; 3D view; checkCIF report

## Figures and Tables

**Fig. 1.**
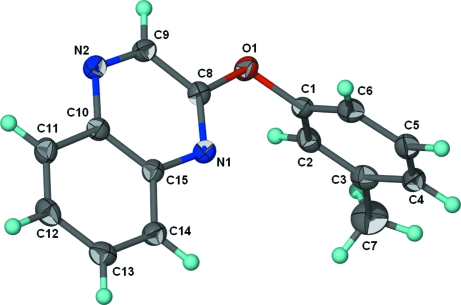
Displacement ellipsoid plot (Barbour, 2001) of C15H12N2O at the 70% probability level; hydrogen atoms are drawn as spheres of arbitrary radius.

## supplementary crystallographic information

### Comment

(type here to add)

### Experimental

*m*-Cresol (0.54 g, 5 mmol) was dissolved in a small volume of water containing potassium hydroxide (0.20 g, 5 mmol). The mixture was heated to remove the water to give a brown compound. The compound and 2-chloroquinoxaline (0.82, g, 5 mmol) were heated in THF (15 ml) for 8 h. The mixture was in 1 N sodium hydroxide; the aqueous solution was extracted with dichloromethane. The organic phase was dried over sodium sulfate. Evaporation of the solvent gave a yellow product, which was was washed with chloroform to remove impurities. Crystals were obtained upon recrystallization from an ethyl acetate/hexane mixture.

### Refinement

Carbon-bound H-atoms were placed in calculated positions (C—H 0.95–0.98 Å) and were included in the refinement in the riding model approximation, with *U*(H) fixed at 1.2–1.5*U*(C).

### Crystal data

**Table:**

C_15_H_12_N_2_O	*F*_000_ = 992
*M**_r_* = 236.27	*D*_x_ = 1.326 Mg m^−^^3^
Monoclinic, *C*2/*c*	Mo *K*α radiation λ = 0.71073 Å
Hall symbol: -C 2yc	Cell parameters from 756 reflections
*a* = 18.5958 (5) Å	θ = 2.7–24.6º
*b* = 7.0710 (1) Å	µ = 0.09 mm^−^^1^
*c* = 19.4821 (5) Å	*T* = 100 (2) K
β = 112.487 (1)º	Block, colorless
*V* = 2366.94 (9) Å^3^	0.40 × 0.15 × 0.10 mm
*Z* = 8	

### Data collection

**Table:**

Bruker SMART APEX diffractometer	2179 reflections with *I* > 2σ(*I*)
Radiation source: fine-focus sealed tube	*R*_int_ = 0.025
Monochromator: graphite	θ_max_ = 27.5º
*T* = 100(2) K	θ_min_ = 2.3º
ω scans	*h* = −24→24
Absorption correction: None	*k* = −9→9
8036 measured reflections	*l* = −25→25
2708 independent reflections	

### Refinement

**Table:**

Refinement on *F*^2^	Secondary atom site location: difference Fourier map
Least-squares matrix: full	Hydrogen site location: inferred from neighbouring sites
*R*[*F*^2^ > 2σ(*F*^2^)] = 0.040	H-atom parameters constrained
*wR*(*F*^2^) = 0.112	*w* = 1/[σ^2^(*F*_o_^2^) + (0.0586*P*)^2^ + 1.0233*P*] where *P* = (*F*_o_^2^ + 2*F*_c_^2^)/3
*S* = 1.04	(Δ/σ)_max_ = 0.001
2708 reflections	Δρ_max_ = 0.29 e Å^−^^3^
164 parameters	Δρ_min_ = −0.26 e Å^−^^3^
Primary atom site location: structure-invariant direct methods	Extinction correction: none

### Fractional atomic coordinates and isotropic or equivalent isotropic displacement parameters (Å^2^)

**Table:**

	*x*	*y*	*z*	*U*_iso_*/*U*_eq_	
O1	0.67552 (5)	0.84219 (13)	0.54655 (5)	0.0215 (2)	
N1	0.58519 (6)	0.74119 (14)	0.59395 (5)	0.0174 (2)	
N2	0.47213 (6)	0.77607 (15)	0.44719 (6)	0.0200 (2)	
C1	0.73297 (7)	0.83475 (17)	0.61927 (7)	0.0179 (3)	
C2	0.78445 (7)	0.68518 (17)	0.63734 (7)	0.0202 (3)	
H2	0.7802	0.5894	0.6018	0.024*	
C3	0.84288 (7)	0.67583 (18)	0.70839 (7)	0.0223 (3)	
C4	0.84907 (7)	0.82240 (19)	0.75792 (7)	0.0215 (3)	
H4	0.8893	0.8193	0.8060	0.026*	
C5	0.79743 (7)	0.97296 (18)	0.73822 (7)	0.0220 (3)	
H5	0.8027	1.0723	0.7727	0.026*	
C6	0.73806 (7)	0.97912 (17)	0.66841 (7)	0.0206 (3)	
H6	0.7018	1.0804	0.6548	0.025*	
C7	0.89521 (8)	0.5050 (2)	0.73175 (9)	0.0364 (4)	
H7A	0.9393	0.5328	0.7781	0.055*	
H7B	0.9145	0.4736	0.6929	0.055*	
H7C	0.8658	0.3977	0.7395	0.055*	
C8	0.60151 (7)	0.79686 (16)	0.53826 (7)	0.0174 (3)	
C9	0.54531 (7)	0.81523 (17)	0.46401 (7)	0.0192 (3)	
H9	0.5618	0.8570	0.4260	0.023*	
C10	0.45167 (7)	0.71606 (16)	0.50459 (7)	0.0176 (3)	
C11	0.37355 (7)	0.66926 (17)	0.48997 (7)	0.0218 (3)	
H11	0.3356	0.6792	0.4408	0.026*	
C12	0.35219 (7)	0.60961 (18)	0.54625 (7)	0.0230 (3)	
H12	0.2994	0.5791	0.5362	0.028*	
C13	0.40826 (7)	0.59334 (17)	0.61903 (7)	0.0219 (3)	
H13	0.3930	0.5512	0.6577	0.026*	
C14	0.48469 (7)	0.63759 (17)	0.63472 (7)	0.0194 (3)	
H14	0.5219	0.6266	0.6841	0.023*	
C15	0.50814 (7)	0.69927 (16)	0.57774 (7)	0.0163 (3)	

### Atomic displacement parameters (Å^2^)

**Table:**

	*U*^11^	*U*^22^	*U*^33^	*U*^12^	*U*^13^	*U*^23^
O1	0.0167 (4)	0.0313 (5)	0.0162 (4)	−0.0030 (4)	0.0059 (3)	0.0016 (4)
N1	0.0173 (5)	0.0183 (5)	0.0161 (5)	0.0006 (4)	0.0057 (4)	0.0004 (4)
N2	0.0221 (6)	0.0191 (5)	0.0168 (5)	−0.0012 (4)	0.0051 (4)	0.0003 (4)
C1	0.0150 (6)	0.0236 (6)	0.0156 (6)	−0.0040 (5)	0.0063 (5)	0.0018 (5)
C2	0.0197 (6)	0.0207 (6)	0.0228 (6)	−0.0035 (5)	0.0108 (5)	−0.0027 (5)
C3	0.0173 (6)	0.0252 (6)	0.0266 (7)	0.0009 (5)	0.0106 (5)	0.0046 (5)
C4	0.0160 (6)	0.0301 (7)	0.0176 (6)	−0.0044 (5)	0.0055 (5)	0.0027 (5)
C5	0.0240 (7)	0.0248 (6)	0.0201 (6)	−0.0051 (5)	0.0116 (5)	−0.0028 (5)
C6	0.0203 (6)	0.0213 (6)	0.0223 (6)	0.0011 (5)	0.0104 (5)	0.0017 (5)
C7	0.0294 (8)	0.0366 (8)	0.0422 (9)	0.0122 (6)	0.0127 (7)	0.0077 (7)
C8	0.0167 (6)	0.0168 (6)	0.0190 (6)	0.0001 (4)	0.0071 (5)	−0.0010 (5)
C9	0.0220 (7)	0.0197 (6)	0.0161 (6)	−0.0009 (5)	0.0076 (5)	0.0005 (5)
C10	0.0194 (6)	0.0141 (5)	0.0186 (6)	0.0004 (4)	0.0065 (5)	−0.0001 (5)
C11	0.0180 (6)	0.0209 (6)	0.0218 (6)	−0.0007 (5)	0.0023 (5)	0.0011 (5)
C12	0.0178 (6)	0.0227 (6)	0.0291 (7)	−0.0027 (5)	0.0097 (5)	−0.0005 (5)
C13	0.0251 (7)	0.0200 (6)	0.0237 (6)	−0.0011 (5)	0.0128 (5)	0.0004 (5)
C14	0.0213 (6)	0.0190 (6)	0.0180 (6)	0.0003 (5)	0.0074 (5)	0.0003 (5)
C15	0.0178 (6)	0.0133 (5)	0.0172 (6)	0.0010 (4)	0.0060 (5)	−0.0016 (4)

### Geometric parameters (Å, °)

**Table:**

O1—C8	1.3612 (14)	C6—H6	0.9500
O1—C1	1.4119 (14)	C7—H7A	0.9800
N1—C8	1.2947 (16)	C7—H7B	0.9800
N1—C15	1.3764 (15)	C7—H7C	0.9800
N2—C9	1.3018 (15)	C8—C9	1.4302 (17)
N2—C10	1.3787 (16)	C9—H9	0.9500
C1—C6	1.3778 (17)	C10—C11	1.4087 (17)
C1—C2	1.3787 (17)	C10—C15	1.4153 (16)
C2—C3	1.3967 (17)	C11—C12	1.3678 (18)
C2—H2	0.9500	C11—H11	0.9500
C3—C4	1.3905 (19)	C12—C13	1.4071 (18)
C3—C7	1.5080 (18)	C12—H12	0.9500
C4—C5	1.3859 (18)	C13—C14	1.3707 (17)
C4—H4	0.9500	C13—H13	0.9500
C5—C6	1.3863 (17)	C14—C15	1.4082 (17)
C5—H5	0.9500	C14—H14	0.9500
			
C8—O1—C1	116.93 (9)	H7B—C7—H7C	109.5
C8—N1—C15	115.49 (10)	N1—C8—O1	121.51 (11)
C9—N2—C10	116.58 (10)	N1—C8—C9	124.08 (11)
C6—C1—C2	122.30 (11)	O1—C8—C9	114.41 (10)
C6—C1—O1	119.61 (11)	N2—C9—C8	121.63 (11)
C2—C1—O1	118.02 (11)	N2—C9—H9	119.2
C1—C2—C3	119.36 (11)	C8—C9—H9	119.2
C1—C2—H2	120.3	N2—C10—C11	119.44 (11)
C3—C2—H2	120.3	N2—C10—C15	120.93 (11)
C4—C3—C2	118.62 (11)	C11—C10—C15	119.63 (11)
C4—C3—C7	121.00 (12)	C12—C11—C10	120.23 (12)
C2—C3—C7	120.30 (12)	C12—C11—H11	119.9
C5—C4—C3	121.04 (11)	C10—C11—H11	119.9
C5—C4—H4	119.5	C11—C12—C13	120.17 (12)
C3—C4—H4	119.5	C11—C12—H12	119.9
C6—C5—C4	120.22 (12)	C13—C12—H12	119.9
C6—C5—H5	119.9	C14—C13—C12	120.76 (12)
C4—C5—H5	119.9	C14—C13—H13	119.6
C1—C6—C5	118.40 (12)	C12—C13—H13	119.6
C1—C6—H6	120.8	C13—C14—C15	120.15 (11)
C5—C6—H6	120.8	C13—C14—H14	119.9
C3—C7—H7A	109.5	C15—C14—H14	119.9
C3—C7—H7B	109.5	N1—C15—C14	119.66 (11)
H7A—C7—H7B	109.5	N1—C15—C10	121.29 (11)
C3—C7—H7C	109.5	C14—C15—C10	119.05 (11)
H7A—C7—H7C	109.5		
			
C8—O1—C1—C6	−77.38 (14)	N1—C8—C9—N2	−0.01 (19)
C8—O1—C1—C2	105.36 (13)	O1—C8—C9—N2	179.64 (11)
C6—C1—C2—C3	1.58 (18)	C9—N2—C10—C11	−179.26 (11)
O1—C1—C2—C3	178.76 (10)	C9—N2—C10—C15	0.17 (17)
C1—C2—C3—C4	−2.44 (18)	N2—C10—C11—C12	−179.95 (11)
C1—C2—C3—C7	174.40 (12)	C15—C10—C11—C12	0.61 (18)
C2—C3—C4—C5	1.48 (18)	C10—C11—C12—C13	−0.45 (19)
C7—C3—C4—C5	−175.34 (12)	C11—C12—C13—C14	0.31 (19)
C3—C4—C5—C6	0.41 (18)	C12—C13—C14—C15	−0.33 (19)
C2—C1—C6—C5	0.31 (18)	C8—N1—C15—C14	−179.98 (11)
O1—C1—C6—C5	−176.82 (10)	C8—N1—C15—C10	0.31 (16)
C4—C5—C6—C1	−1.31 (18)	C13—C14—C15—N1	−179.24 (11)
C15—N1—C8—O1	−179.77 (10)	C13—C14—C15—C10	0.48 (18)
C15—N1—C8—C9	−0.15 (17)	N2—C10—C15—N1	−0.33 (17)
C1—O1—C8—N1	−3.05 (16)	C11—C10—C15—N1	179.09 (11)
C1—O1—C8—C9	177.29 (10)	N2—C10—C15—C14	179.95 (11)
C10—N2—C9—C8	0.00 (17)	C11—C10—C15—C14	−0.62 (17)
